# Talaromycosis in a Patient on Nintedanib for Interstitial Lung Disease

**DOI:** 10.7759/cureus.7215

**Published:** 2020-03-08

**Authors:** Anantha Sriharsha Madgula, Brian R Covello, Meghana Singh, Arundati Rao, Jim C Lee

**Affiliations:** 1 Internal Medicine, University of Connecticut School of Medicine, Farmington, USA; 2 Internal Medicine, University of Connecticut Health Center, Hartford, USA; 3 Internal Medicine, University of Connecticut, Hartford, USA; 4 Pathology, Hartford Hospital, Hartford, USA

**Keywords:** talaromycosis, penicilliosis, nintedanib, interstitial lung disease

## Abstract

Talaromycosis is a fungal infection caused by *Talaromyces *sp. that is predominantly prevalent in patients with acquired immunodeficiency syndrome in the United States. It is also rarely seen in other individuals who are otherwise immunosuppressed. With the advent of immunotherapy and increasing usage of these novel agents in treating several conditions, the prevalence of talaromycosis may increase, especially in people from endemic regions who might harbor a dormant infection. Clinical presentation is non-specific with respiratory symptoms such as shortness of breath, cough, or even fever that can delay the diagnosis. Little is known about the exact pathogenesis of the condition, and management is largely based on anecdotal evidence and small-sized studies. We present the case of an individual on nintedanib, a tyrosine kinase inhibitor that blocks fibroblast growth factor receptor and used for the treatment of interstitial lung disease, who was diagnosed with talaromycosis.

## Introduction

Talaromycosis, formerly known as penicilliosis, is a fungal infection caused by *Talaromyces *sp. The dimorphic fungus was first isolated from *Rhizomys sinensis* (bamboo rat) in Vietnam in 1956, and classically causes opportunistic fungal infection in HIV-infected individuals who reside or travel to Southeast Asia [1]. *T*alaromyces* marneffei* was not recognized as a potential infection until a 1959 laboratory experiment conducted in France proved it was possible to acquire the pathogen [2]. It was not until 1973 that the first patient infection was reported in the literature [2].

Transmission of infection occurs through inhalation of conidia or skin contact from an environmental reservoir, and the organism can affect the respiratory tract, gastrointestinal tract, skin, mucosa, or neurological system. It is unknown whether person-to-person transmission occurs [1,2]. Because phagocytic cells defend against this infection, immunocompetent patients present with suppurative reactions while immunocompromised patients present with necrotizing infections [1]. The typical talaromycosis infection has a broad clinical presentation and may manifest as weight loss, fever, epidermal lesions, lymphadenopathy, hepatosplenomegaly, or respiratory symptoms. Infection of the respiratory tract presents with cough, fever, dyspnea, and chest pain. Pulmonary manifestations on chest radiography may include diffuse reticulonodular, localized alveolar, or diffuse alveolar infiltrates [1,3].

With the advent of antiretrovirals, talaromycosis is now a relatively rare AIDS-defining infection, and there have only been very few reported cases in non-endemic immunocompetent individuals [1,4]. In immunocompetent patients, talaromycosis appears to occur more often in those with other systemic diseases such as systemic lupus erythematosus (SLE) or sarcoidosis [1,2]. However, it is difficult to judge this prevalence, given the low number of overall case reports. If diagnosed in time, three out of four patients with talaromycosis have favorable outcomes [1,2]. We present the case of an HIV-negative patient who presented with talaromycosis on nintedanib therapy, a tyrosine kinase inhibitor, for interstitial lung disease.

## Case presentation

**Figure 1:**
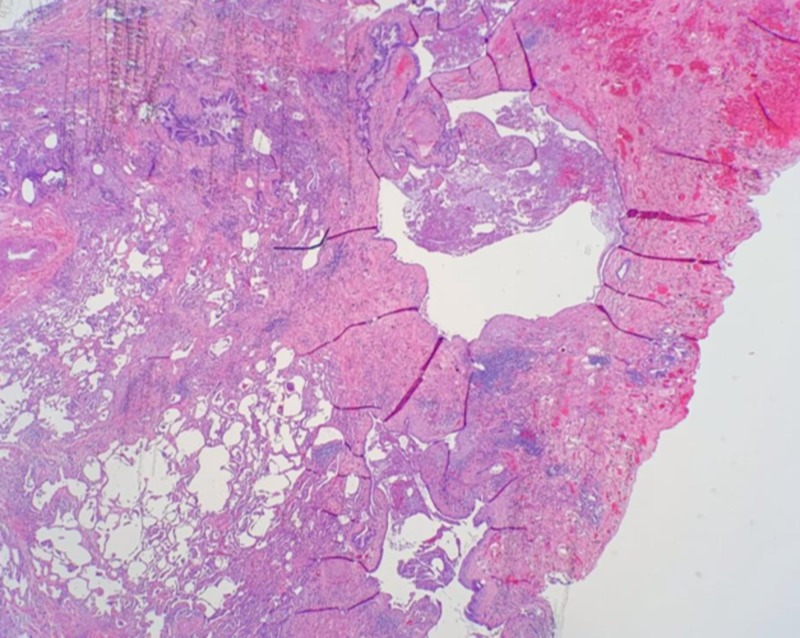
Low power field (2X). The histologic features show extensive subpleural fibrosis with prominent cystic changes (“microscopic honeycombing”) on the top right, compared with the relatively normal-sized alveolar spaces on the left lower side.

**Figure 2:**
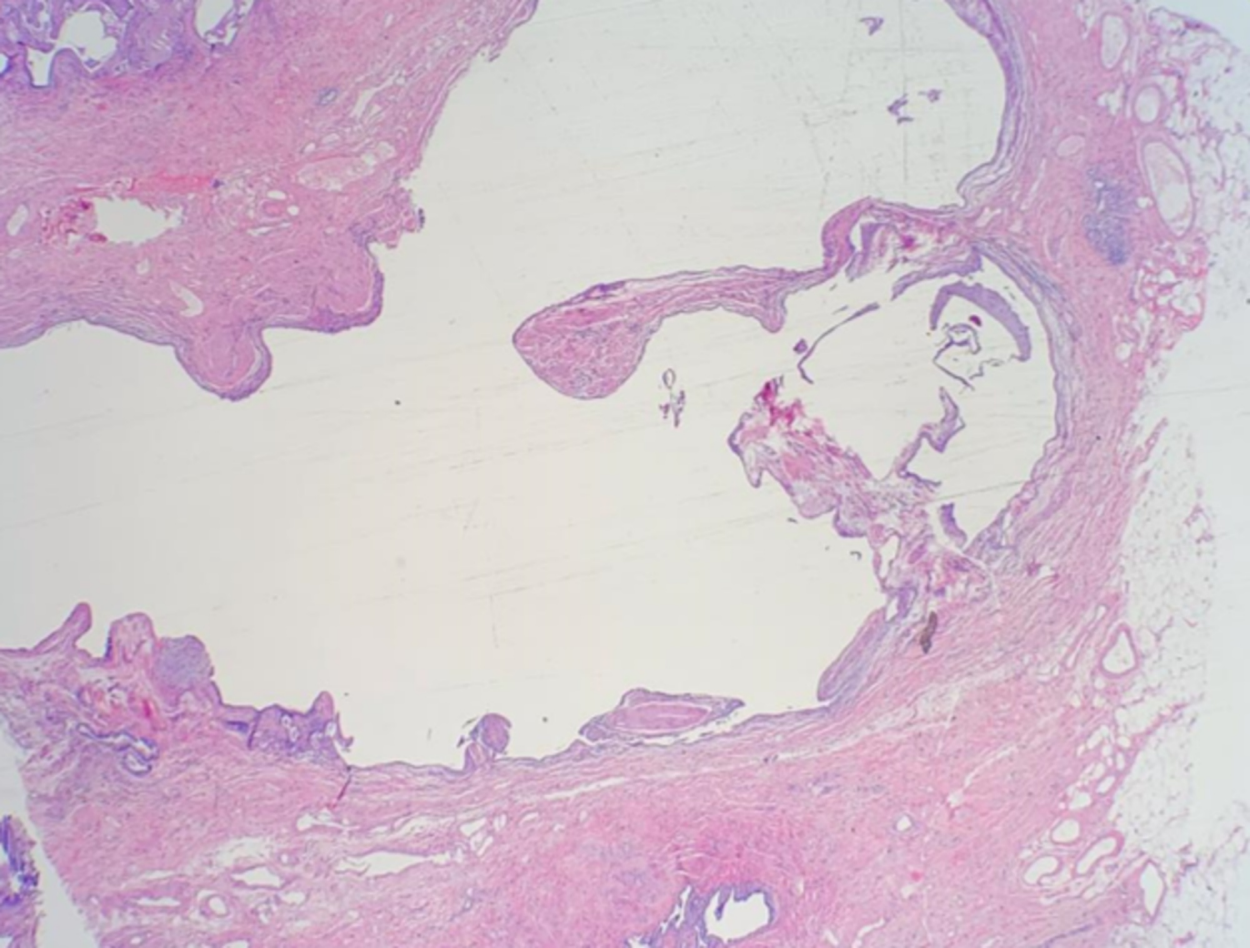
Low power field (2X). Extensive subpleural fibrosis with prominent cystic changes with pleural fibrofatty adhesions/metaplasia.

Our patient is a 72-year-old gentleman with a past medical history of paroxysmal atrial fibrillation, hypertension, and diabetes mellitus, who was recently diagnosed with interstitial lung disease. He was originally from Pakistan, immigrated to the United States in 2006 with notable travel history to the United Arab Emirates several years back. Two months prior to this admission, he was sent to the emergency room from an urgent care clinic when he was found to be in new-onset atrial fibrillation. Workup at that time included a chest X-ray which was abnormal, and hence a CT scan of the chest followed which showed interstitial pneumonia and a round soft tissue density in the right upper lobe surrounded by honeycombing reported as possibly a mycetoma with prominent mediastinal lymph nodes. An elective video-assisted thoracoscopic surgery (VATS) lung biopsy was done, and histopathology showed features consistent with usual interstitial pneumonia (Figures 1, 2). He was tested for HIV and tuberculosis, which were negative. He followed with his pulmonologist and was started on nintedanib therapy as an outpatient.

**Figure 3:**
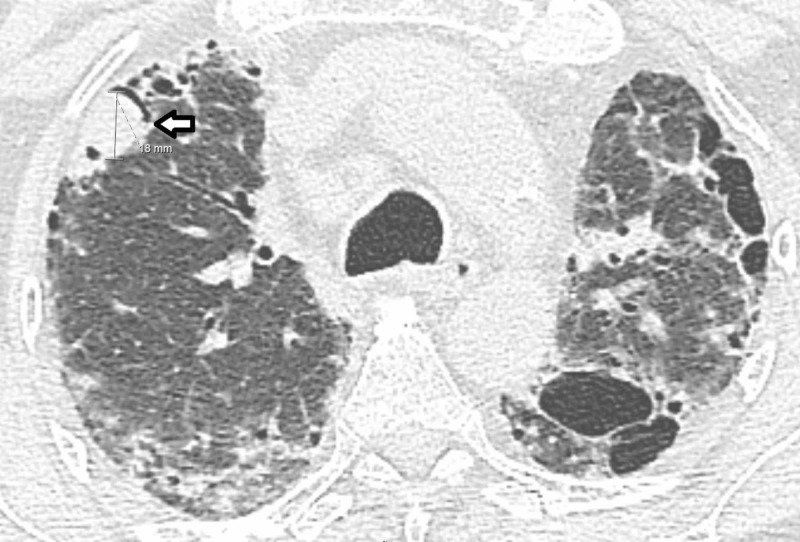
CT scan of chest without contrast demonstrating a mycetoma (left arrow) along with diffuse fibrotic changes.

He subsequently presented to our hospital with dyspnea on exertion that had gradually worsened over two weeks. He was noted to have a significant increase in oxygen requirement. He was hemodynamically stable and afebrile. Physical examination was significant for respiratory distress and diffuse crackles bilaterally. Initial labs were significant for hemoglobin of 11.9 g/dL with a hematocrit of 36.4%, a white blood cell count of 11,400 cells/mm^3^, and a CD4 cell count of 513 cells/mm^3^. Repeat CT scan of his chest without contrast showed findings consistent with interstitial lung disease, now including both microcystic and macrocystic honeycombing more pronounced in the lung bases. A rounded soft tissue density was noted within one of these macrocystic changes in the right upper lobe, measuring up to 1.8 cm (Figure 3), slightly increased from prior where it measured up to 1.3 cm. Superimposed on chronic findings, there was evidence of new bibasilar airspace disease, left greater than right without evidence of pleural effusion or pneumothorax. Two of the fungal cultures sent from his VATS biopsy were growing *Talaromyces* sp. at this time. The patient was started on vancomycin, cefepime, azithromycin, and liposomal amphotericin while premedicating with normal saline and acetaminophen. However, after receiving the amphotericin, the patient developed a hypersensitivity reaction twice despite premedication and had to be switched to voriconazole. His respiratory status continued to worsen with high oxygen requirement necessitating non-invasive positive pressure ventilation, and subsequent intubation. He developed shock with multiorgan failure, and he was transitioned to comfort measures. 

## Discussion

Due to its low incidence, talaromycosis may be a difficult diagnosis for physicians to make and may frequently be missed altogether until the appropriate fungal cultures return. The mortality of this disease can be as high as 91.3% without early diagnosis and appropriate treatment [1]. Relatively little is known about the pathogenesis of *T. marneffei*, and to the best of our knowledge, there are no case reports we could find that associate talaromycosis with interstitial lung disease or nintedanib. To date, talaromycosis has been reported in patients with various autoimmune disorders, including SLE, mixed connective tissue disease, Sjogren's syndrome, primary biliary cirrhosis, primary immune thrombocytopenia, and autoimmune hemolytic anemia [1,2]. In these patients, it is thought that the treatment for these autoimmune diseases, rather than the diseases themselves, may be the exacerbating factor that brings about a systemic talaromyces infection [1,2].

High, prolonged doses of T-cell inhibiting drugs may specifically prime a patient for talaromycosis. Talaromyces infections have taken place in individuals who have received corticosteroids, cyclosporine, azathioprine, tacrolimus, or mycophenolate [2,4]. Our patient was being treated for interstitial lung disease with nintedanib, a potent inhibitor of platelet-derived growth factor receptor, fibroblast growth factor receptor, and vascular endothelial growth factor receptor. While there is no specific mention of nintedanib-associated talaromycosis in the literature, there have been some studies relating novel immunotherapies to this infection [1]. There are reported cases of systemic *T. marneffei* in patients receiving anti-CD20 monoclonal antibodies and kinase inhibitors. Treatment with these agents has a B-cell depleting effect, and it is hypothesized that much like hepatitis B, *T. marneffei* infection may be reactivated through the use of these medications [1,2,4]. With the increasing use of novel immunotherapy agents in the United States, it is imperative for physicians to have a high and reasonable index of suspicion for reactivation or induction of systemic infections.

Management of talaromycosis remains ambiguous, and practices are derived based on expert opinions and small open-labeled studies from endemic regions. Given the rarity of the disease in patients without HIV, no specific guidelines exist for this patient population. Treatment is usually divided into induction therapy and maintenance therapy. Induction is preferred with amphotericin and maintenance with itraconazole [4-6]. An open-labeled non-inferiority trial done by Le et al. in 2017 comparing amphotericin and itraconazole for initial treatment of talaromycosis found that amphotericin was superior to itraconazole in achieving better fungal clearance and lower rates of relapse but had significantly higher rates of transfusion-associated reactions, renal failure, hypokalemia, hypomagnesemia, and anemia [7]. In one observational study, there was an estimated relapse rate of about 33% after the initial response, and hence, maintenance therapy with itraconazole is recommended [5,6]. Intravenous voriconazole, followed by oral voriconazole, was also studied as an alternative to the conventional regimen with good outcomes [5,6].

In our patient, induction therapy with liposomal amphotericin B was started; however, the patient had a hypersensitivity reaction to it despite premedication with intravenous fluids and acetaminophen. At this point, a decision was made to switch the patient to voriconazole due to its better side effect profile.

## Conclusions

As HIV-infected patients have been more appropriately treated, the epidemiology of *T. marneffei* infection continues to evolve. Herein, we present a case report of an HIV-negative patient with known interstitial lung disease on immunomodulating therapy who subsequently developed talaromycosis. There is a strong association of this infection with the immune system, yet the exact pathogenic mechanisms are not entirely known. We cannot make a statement on the causal mechanisms of our patient’s talaromycosis infection, yet we remain suspicious as to the role nintedanib played in modulating our patient’s immune system. More research is needed to elucidate this connection, and case reports such as this remain critical for broadening the knowledge base of the rare infection caused by *T. marneffei*.
